# *In silico* identification of deep-sea fungal alkaloids as potential inhibitors of SARS-CoV-2, Delta and Omicron spikes

**DOI:** 10.2217/fvl-2023-0102

**Published:** 2023-10-26

**Authors:** Abdullah R Alanzi, Mohammad K Parvez, Mohammed S Al-Dosari

**Affiliations:** ^1^Department of Pharmacognosy, College of Pharmacy, King Saud University, Riyadh, 11451, Saudi Arabia

**Keywords:** alkaloids, deep-sea fungi, Delta, molecular docking, Omicron, SARS-CoV-2, spike protein

## Abstract

**Aim:** Virtual screening of deep-sea fungal metabolites against SARS-CoV-2 Delta and Omicron spikes as potential antivirals. **Materials & methods:** Deep-sea fungal alkaloids (n ≥ 150) were evaluated against SARS-CoV-2, Delta and Omicron spikes, using various *in silico* approaches, including Admet scores, physiochemical properties, molecular docking (MD) and MD simulation (150 ns). **Results:** The test alkaloids complied with Admet scores and physiochemical properties within acceptable ranges, and followed Lipinski's rule of five. Of these, *Cladosporium sphaerospermum*-derived cladosin K (tetramate alkaloid) for SARS-CoV-2, *Cystobasidium laryngis*-derived saphenol (phenazine alkaloid) for Delta and *Chaetomium globosum*-derived chaetoglobosin E (quinoline alkaloid) for Omicron were identified as potential spike-inhibitors. **Conclusion:** Our data therefore, strongly warrants further experimental validations of cladosin K, saphenol and chaetoglobosin E, especially against the Omicron and Delta spikes.

## Tweetable abstract

For the first time, this study reports *in silico* analysis-based identification of deep-sea fungal alkaloids cladosin K, saphenol and chaetoglobosin E as potential spike-inhibitors of SARS-CoV-2, Delta and Omicron, respectively.

The COVID-19 pandemic caused by the novel SARS-CoV-2 has greatly affected over 765 million people in the world, with >6.9 million deaths [1,2]. Generally, COVID-19 manifests as cough, fever, headache and breathlessness, which may further progress to mild-to-fatal pneumonia in a proportion of infected cases [2]. Historically, within the first 2 years of the pandemic, outstanding knowledge on its epidemiology, clinical presentations and immunobiology was gained. As a quick response, several treatment and preventive strategies, most remarkably, new effective vaccines were licensed to immunize the world population. Notably, though several repurposed drugs are currently in the final stages of trials or have been granted approval for clinical use [1,3], there are no approved anti-SARS-CoV-2 drugs.

SARS-CoV-2 is an RNA virus that encodes four structural and several nonstructural proteins that are crucial for the viral life cycle and host-pathogenesis [4,5]. The SARS-CoV-2 spike (S) protein's structural subunit ‘S1’ binds to the cell receptor ACE2 via the receptor binding domain (RBD), while the subunit ‘S2’ plays a role in membrane fusion [6]. Notably, in the past 2 years, the evolutionary acquired genetic mutations in SARS-CoV-2 ‘S-RBD’ have led to the emergence of its highly transmissible and pathogenic ‘variants of concern (VOC)’ named as Alpha, Beta, Gamma, Delta and Omicron [7]. Therefore, cases of SARS-CoV-2 reactivation and VOCs re-infections in response to waning natural immunity or diminishing vaccine-protection remain a bottle-neck in the complete control of COVID-19 [8]. In view if this, the Spike ‘RBD’ has been widely investigated as a potential target for developing effective anti-SARS-CoV-2 drugs.

Several natural or plant products rich in bioactive secondary metabolites such as alkaloids, flavonoids, saponins, sterols, tannins, coumarins and polyphenolics have been extensively reported for their marked antiviral properties against several pathogenic viruses [9]. In line with this, several *in silico* screening and *in vitro* experimental studies have suggested potential natural compounds and their synthetic analogues for target-specific activities against SARS-CoV-2 proteins [10–13]. Very recently, our *in silico* study targeting Spike protein has identified four plant metabolites catechin, orientin, obetrioside and neridienone as potential inhibitors of Omicron and Delta variants [14]. Furthermore, though scores of pharmacologically important marine products from algae, sponges or else have been reported [15,16], deep-sea fungal metabolites still remain understudied. According to an estimation, of about 24,500 known marine-derived metabolites, only 500 belong to deep-sea fungi [17,18]. Also, there are very limited studies on *in silico* structure–activity elucidations of marine-derived compounds for their *in vitro* or *in vivo* bioactivities. Therefore, in the present study, we have performed structure-activity based screening of deep-sea fungal metabolites against spike proteins of SARS-CoV-2 Delta and Omicron variants toward developing novel, efficacious and safe antiviral drugs.

## Materials & methods

### Ligand & protein preparations

The 3D structures of over 150 alkaloids from deep-sea fungi were retrieved from the PubChem database (https://pubchem.ncbi.nlm.nih.gov/). The ligand viracept or nelfinavir (PDB ID: 2Q64) was used as positive control, while SSAYA10-001 (PubChem ID: 2807230) served as negative control. All ligands were prepared through LigPrep in Maestro [19], and the protonation states were determined. The geometries of all ligands were optimized, and for each ligand, 32 conformers with different protonation states were generated. The OPLS_2005 forcefield was used to minimize the energy of the ligands [20] and was finally saved in ‘.mae’ format for molecular docking.

The crystal structures of SARS-CoV-2 spike (PDB ID: 6M0J), Omicron spike (PDB ID: 7T9L) and Delta spike (PDB ID: 7V8B) proteins (targets) were retrieved from the Protein Data Bank [21] and prepared by the Protein Preparation Wizard in Schrodinger Maestro [22]. During target protein preparations, different steps, i.e., addition of hydrogens, creation of disulfide bonds, zero order bonds to metals and assigning bond orders, were performed. Further, any additional or unwanted ligands and water molecules were removed, and hydrogen bonds were optimized at pH 7.0 by using PROPKA as described elsewhere [23]. Finally, the energy of the proteins was minimized by using the OPLS_2005 forcefield [20]. Protein preparation was followed by generating a 3D grid at the reported binding pocket of the spike proteins in order to conduct site-specific docking.

### Molecular docking analysis

The prepared ligands were docked at binding sites against the three spike proteins using the glide docking module in standard precision mode [24]. Based on the glide scores of the docked ligands, analysis and selection were performed.

### Analysis of physicochemical properties

The physicochemical properties such as molecular weight (MW), hydrogen bond donors (HBDs), hydrogen bond acceptors (HBAs), octanol-water partition coefficient (LogP) and polar surface area (PSA) of the selected ligands were calculated by using the QikProp in Maestro [25].

### Molecular dynamic simulation study

Following the docking analysis, the top compound for each target protein showing binding affinity higher than the positive control was selected for the protein–ligand complex stability analysis at 150 ns long molecular dynamic (MD) simulation using VMD [26] and NAMD [27] tools. Therein, Ambertools 21 was used to generate the input files for simulation [28], whereas Antechamber was utilized to produce the ligand topology files, and the missing hydrogens were added in LeaP program [29]. The ligands were parameterized using the AM1-BCC (Austin Model 1 Bond Charge Correction) method for calculating Gasteiger charges. Then, a solvation box of 10 Å containing the TIP3P water model was used to solvate each ligand–protein complex [30]. Counter Na+ and Cl- ions were added to the systems to neutralize them. The ff14SB and GAFF forcefields were used to generate the parameters of protein and ligand, respectively [31]. The solvated systems were equilibrated for 20 ps after the collisions were eliminated by a minimization phase, and three additional 20 ps equilibrations at 200, 250 and 300 K followed. The equilibration at different temperatures (200 K, 250 K and 300 K) was conducted to investigate the system's behavior across a range of temperature conditions. This approach allows for a comprehensive exploration of the system's phase space and provides insights into its thermodynamic properties and behavior at varying energy levels, which can be essential for a more thorough understanding of the system's dynamics. Then, a 150 ns simulation was performed on the prepared systems at 310 K temperature and 1 atmosphere pressure using NPT ensemble. The hydrogen bonds were constrained using the SHAKE algorithm while the electrostatic charges of non-bonded interactions were calculated using the particle mesh Ewald method. The MD trajectories were stored every 2 ps. The trajectories were examined using the R package [32] and CPPTRAJ [33].

## Results

### Structure-activity prediction of the ligands

Prior to the molecular docking, the efficacy of the docking tool was validated by area under curve study of Receiver operating curve. A dataset containing active and decoy compounds was used for the validation of the tool. Upon analysis, it was observed that the AUC value was 0.75, indicating that the tool was not producing false-positive results. The ROC curve of the validation protocol is shown in Supplementary Figure 1. Further, in the absence of a co-crystalized ligand in PDB, the co-crystalized ligand of SARS-CoV-2 Mpro (PDB ID: 6LU7) was used for re-docking validation step. The co-crystal peptide was extracted and prepared for docking. The prepared co-crystalized peptide was docked to the same site, and the root mean square deviation (RMSD) of docked and co-crystalized ligand was calculated to be 0.22. The minor deviations show the better performance of the Glide tool. The docked pose of the co-crystalized ligand along with reference pose can be seen in Supplementary Figure 2. The virtually-screening of SARS-CoV-2, Omicron and Delta spike proteins performed against deep-sea fungal alkaloids of various sub-classes showed good binging affinities (Supplementary Figure 3). Following molecular docking analysis of the Spike proteins of SARS-CoV-2 (Supplementary Table 2), Delta (Supplementary Table 3), and Omicron (Supplementary Table 4), all docked ligands were ranked based on their glide scores. Of these, the best inhibitors of SARS-CoV-2, Omicron and Delta Spikes were *Cladosporium sphaerospermum*-derived cladosin K (-5.64 kcal/mol), *Cystobasidium laryngis*-derived saphenol (-4.01 kcal/mol) and *Chaetomium globosum*-derived chaetoglobosin E (-4.22 kcal/mol), respectively (Table 1). The estimated scores for the docked viracept were -5.04, -4.52 and -5.30 kcal/mol, respectively. Further, those with binding affinities higher than control-ligand were subjected to binding pose analysis in their respective complexes.

**Table 1.** The estimated physiochemical properties and molecular docking scores of the top three potential inhibitors of SARS-CoV-2, Delta and Omicron spike proteins.

Spike protein (target)	Best compounds (ligand)	MW (g/mol)	HBD	HBA	QPlogP_o/w_	PSA	RO5 (Violations)	Glide score (kcal/mol)
SARS-CoV-2	Cladosin K	419.522	4	5	4.191	101.703	0	–5.64
	Viracept^†^	5676.8	4	6	3.87	127.20	1	–5.04
Delta	Saphenol	268.315	2	5	2.483	61.851	0	–4.01
	Viracept^†^	5676.8	4	6	3.87	127.20	1	–4.52
Omicron	Chaetoglobosin E	530.663	3	8	3.367	132.078	1	–4.22
	Viracept^†^	5676.8	4	6	3.87	127.20	1	–5.30

† Positive control.

HBA: Hydrogen bond acceptor; HBD: Hydrogen bond donor; MW: Molecular weight; PSA: Polar surface area.

### Physicochemical properties prediction of the ligands

The predicted physicochemical properties of the deep-sea fungal alkaloids (Supplementary Table 1), including the top selected inhibitors of SARS-CoV-2, Delta, and Omicron spikes (Table 1) were within the acceptable ranges for MW (<500 g/mol), HBA (<10), HBD (<5) and LogP (<5). Overall, except for a few, all ligands met the criteria of Lipinski's rule of five.

### Binding pose prediction of the complexed ligands

**Figure 1.**
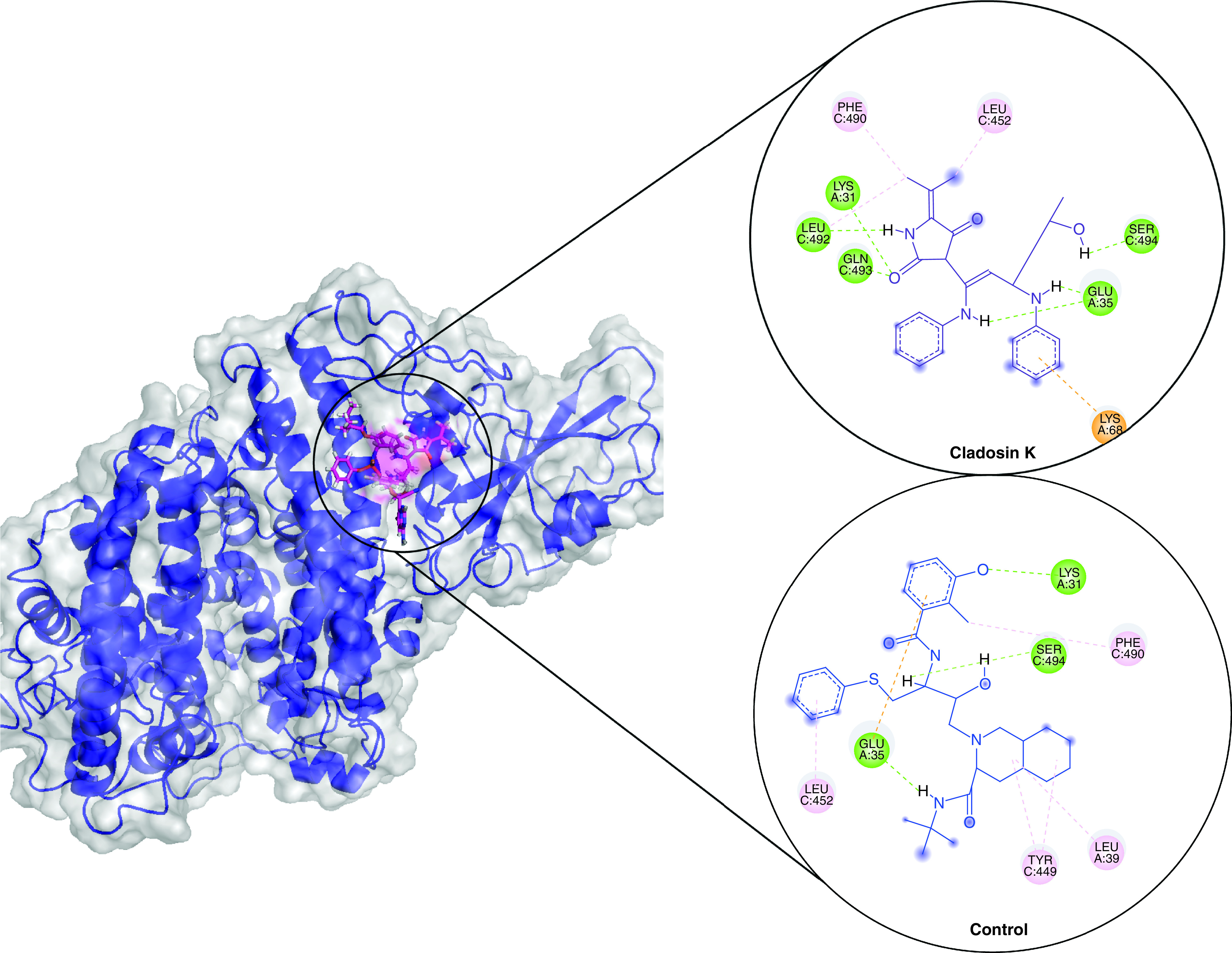
Molecular docking showing binding interactions of cladosin K and viracept with SARS-CoV-2 spike. The interactions are shown in different colors. Hydrogen bonding (green), hydrophobic (magenta), Pi-Cation (orange).

**Figure 2.**
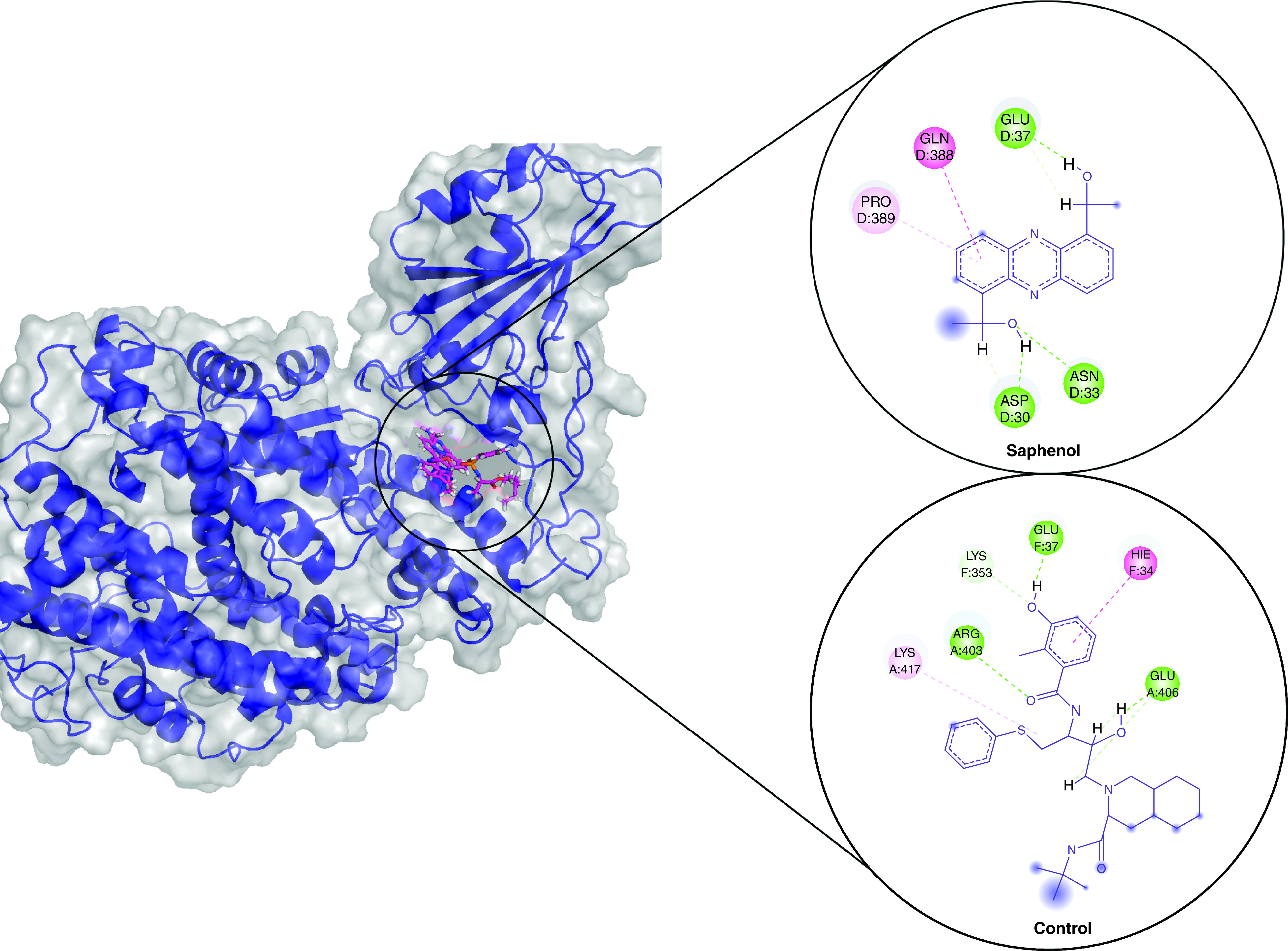
Molecular docking showing binding interactions of saphenol and viracept with Omicron variant spike. The interactions are shown in different colors. Hydrogen bonding (green), hydrophobic (magenta), Pi-Cation (orange).

**Figure 3.**
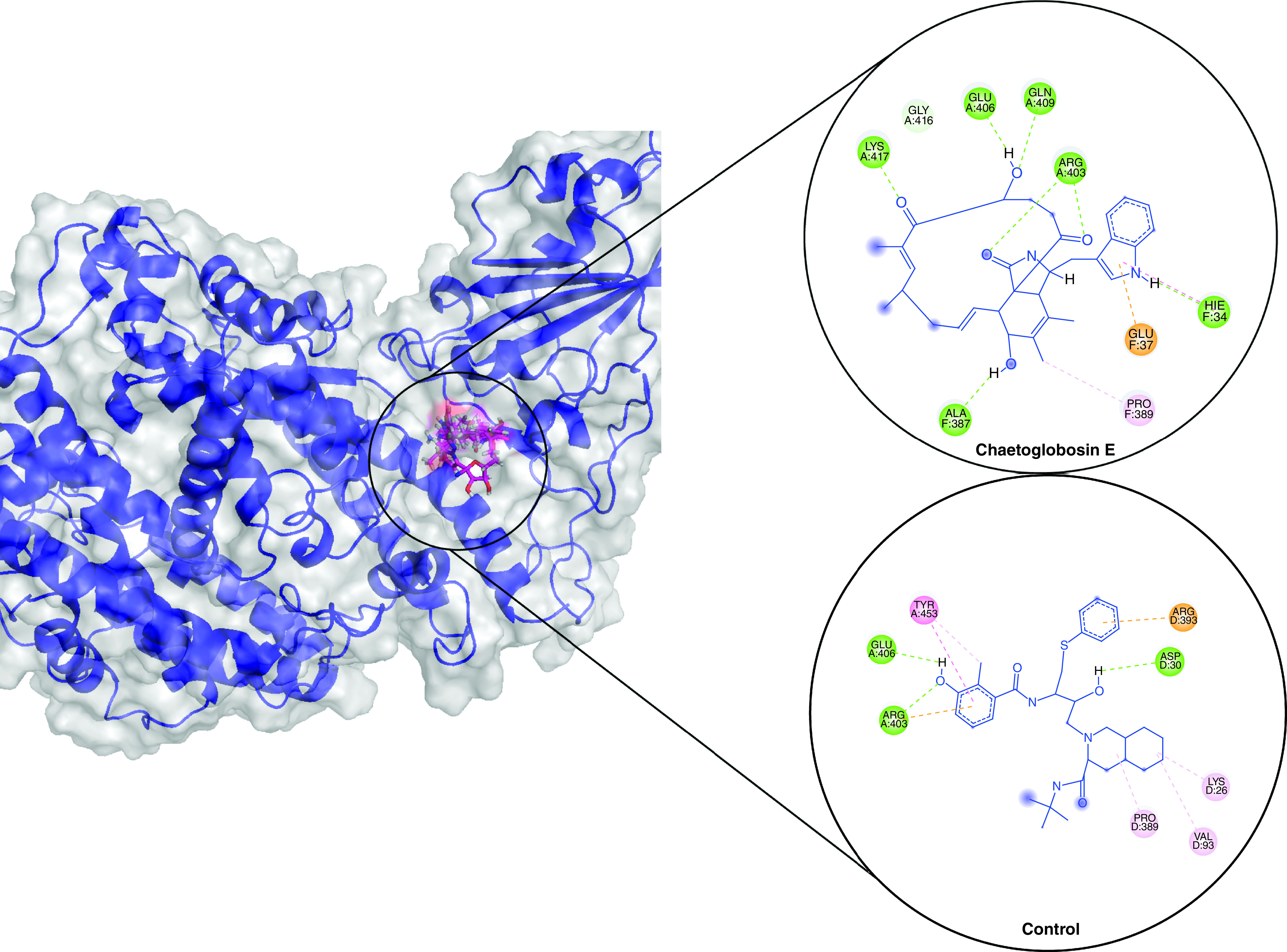
Molecular docking showing binding interactions of chaetoglobosin E and viracept with Delta variant spike. The interactions are shown in different colors. Hydrogen bonding (green), hydrophobic (magenta), Pi-Cation (orange).

Based on the binding poses of the docked ligands, the top compound from each complex was selected for protein–ligand stability analysis and its molecular interactions were compared with the positive control viracept. For the SARS-CoV-2, Cladosin K was selected, which formed five conventional hydrogen bonds with its spike protein residues Lys31, Glu35, Leu492, Gln493 and Ser494 along with other interactions (Figure 1). In comparison, viracept formed three hydrogen bonds with Lys31, Glu35 and Ser494. For Omicron, Saphenol established three hydrogen bonds with its spike residues Asp30, Asn33 and Glu37, including other interactions. Viracept made three hydrogen bonds and two hydrophobic interactions (Figure 2). For Delta, chaetoglobosin E formed six hydrogen bonds with its spike residues His34, Ala387, Arg403, Glu406, Gln409 and Lys417 along with other interaction. Viracept formed three hydrogen bonds with Asp30, Ath403 and Glu406 (Figure 3).

### MD simulation analysis of the selected compounds

The RMSD, root mean square fluctuation (RMSF) and Rg of the three selected compounds complexed with their respective spike proteins were compared with the apo protein structure. Further, the binding free energy values of the complexes were estimated through molecular mechanics with generalized Born and surface area solvation (MM/GBSA) approach and the binding energy per frame was also calculated to find the stability of the complexes during MD simulation.

### Cladosin K & SARS-CoV-2 Spike complex

**Figure 4.**
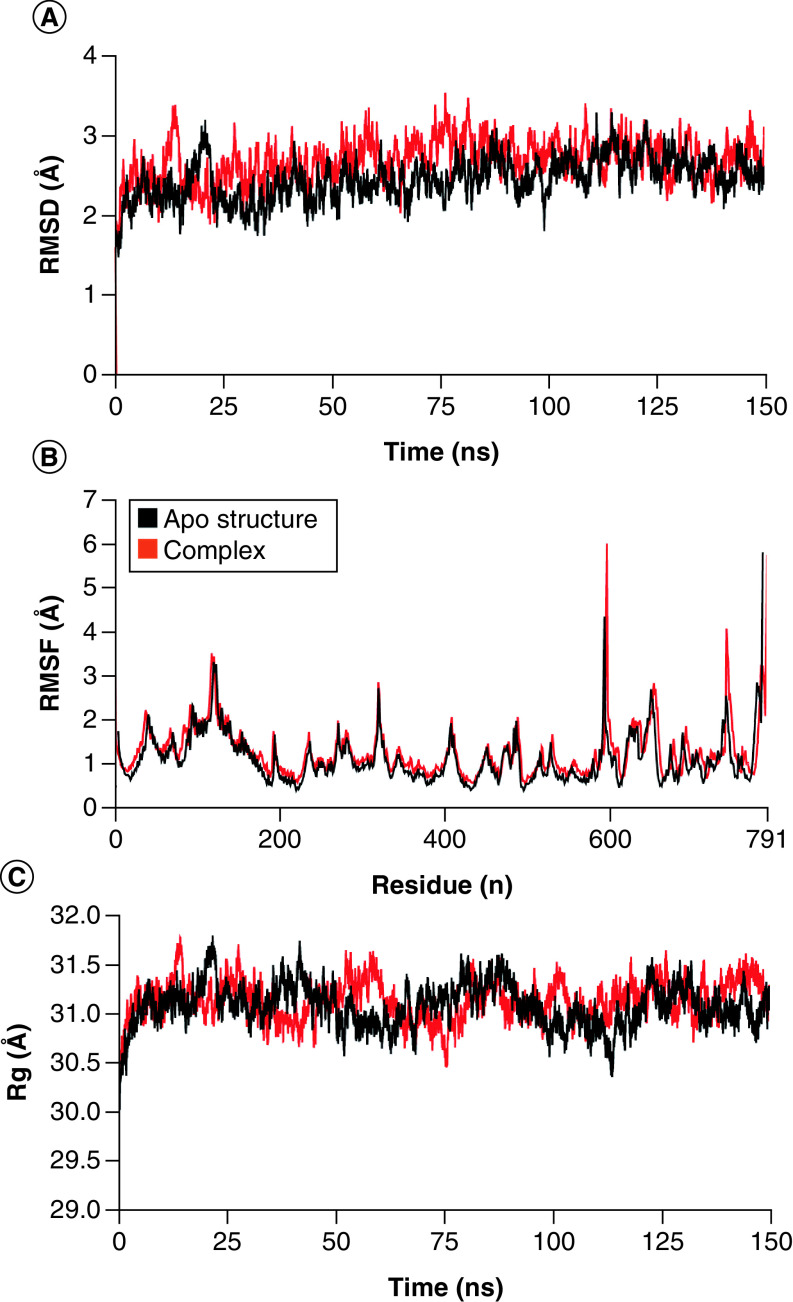

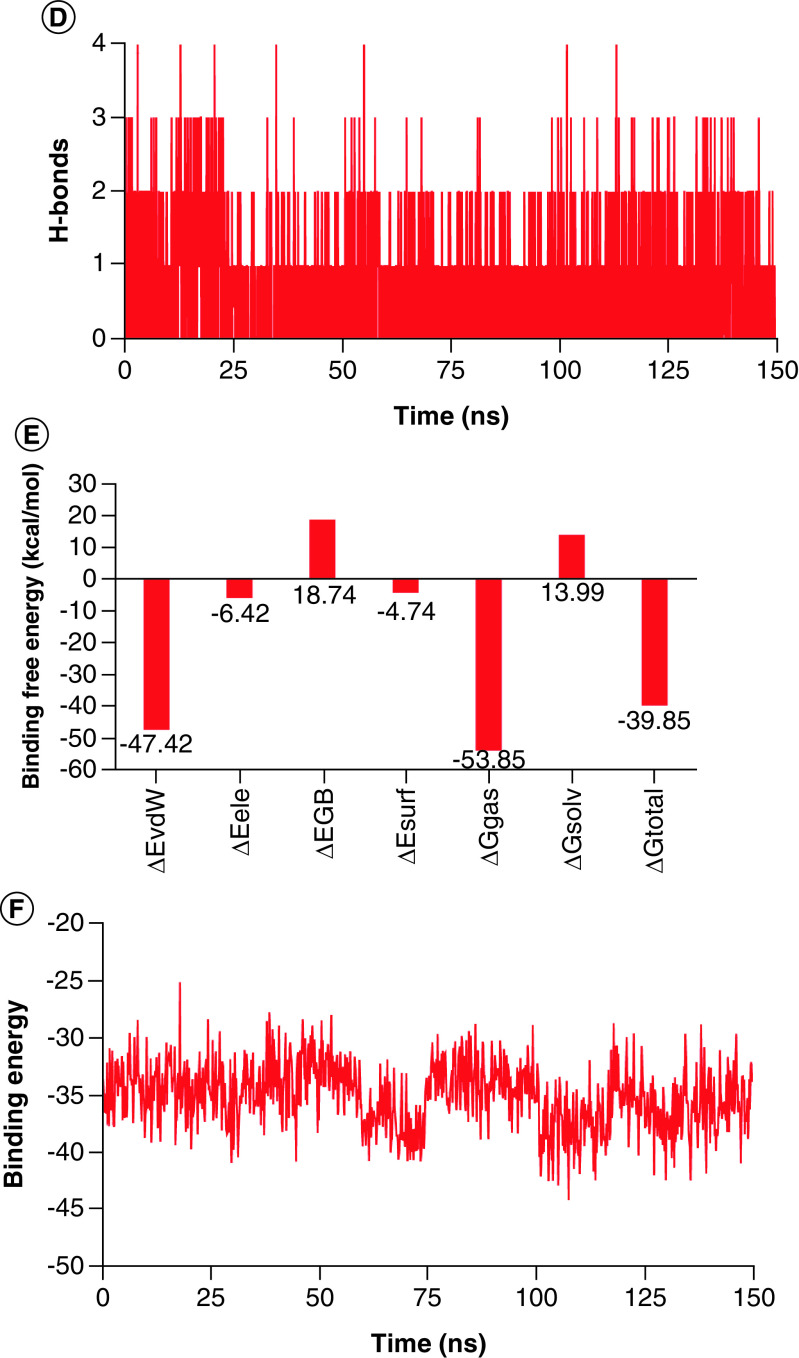
Molecular docking simulation analysis of cladosin K and SARS-CoV-2 Spike complex. **(A)** Root mean square deviation analysis of protein backbone. **(B)** Root mean square fluctuation analysis of protein fluctuations. **(C)** Radius of gyration calculation. **(D)** Hydrogen bonding analysis. **(E)** Molecular mechanics with generalized Born and surface area solvation to calculate binding free energy. **(F)** Binding energy per frame during simulation. RMSD: Root mean square deviation; RMSF: Root mean square fluctuation.

In the cladosin K and SARS-CoV-2 Spike complex, the RMSD of the backbone atoms of spike protein remained in the range of ∼2.5–3 Å throughout the simulation, except for some minor deviations at different time intervals. While there RMSD values attained stability in the range of ∼2.5 Å, that of the apo protein remained in the range of ∼2–2.5 Å after 25 ns (Figure 4A), indicating the protein–ligand stability. The RMSF analysis revealed no major fluctuations in protein residues except for C- and N-termini, and amino acids ranging from 590–610 for the spike and apo structures (Figure 4B). The Rg plot showed that the spikes and apo proteins remained compact when complexed with their ligands during the simulation, which indicated the stability of the proteins in complex form (Figure 4C). The hydrogen bonding analysis showed that the protein and ligand formed a minimum of two hydrogen bonds throughout the simulation (Figure 4D). The total binding free energy (MMGBSA) of the complex was -39.85 kcal/mol (Figure 4E), and the average energy per frames was -35 to -37 kcal/mol (Figure 4F).

### Saphenol & Omicron spike complex

**Figure 5.**
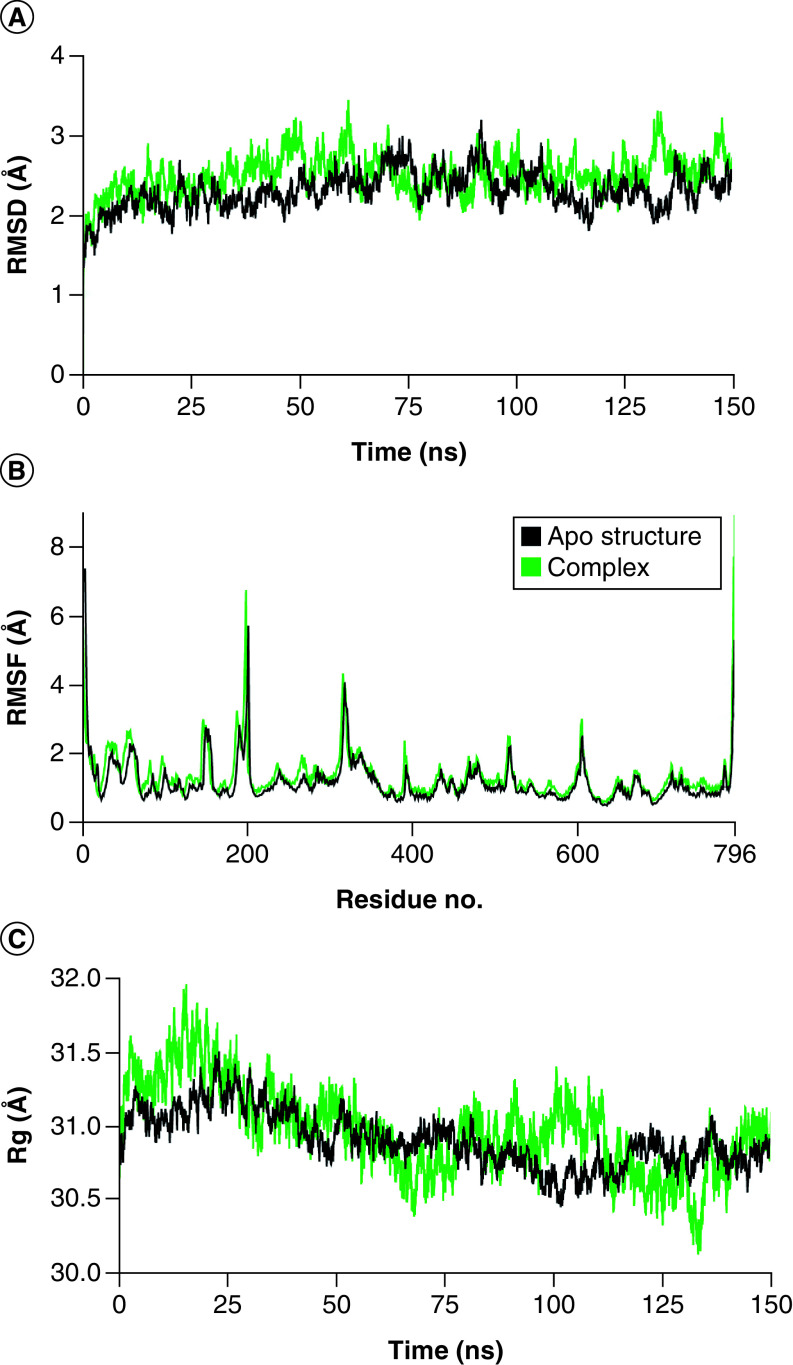

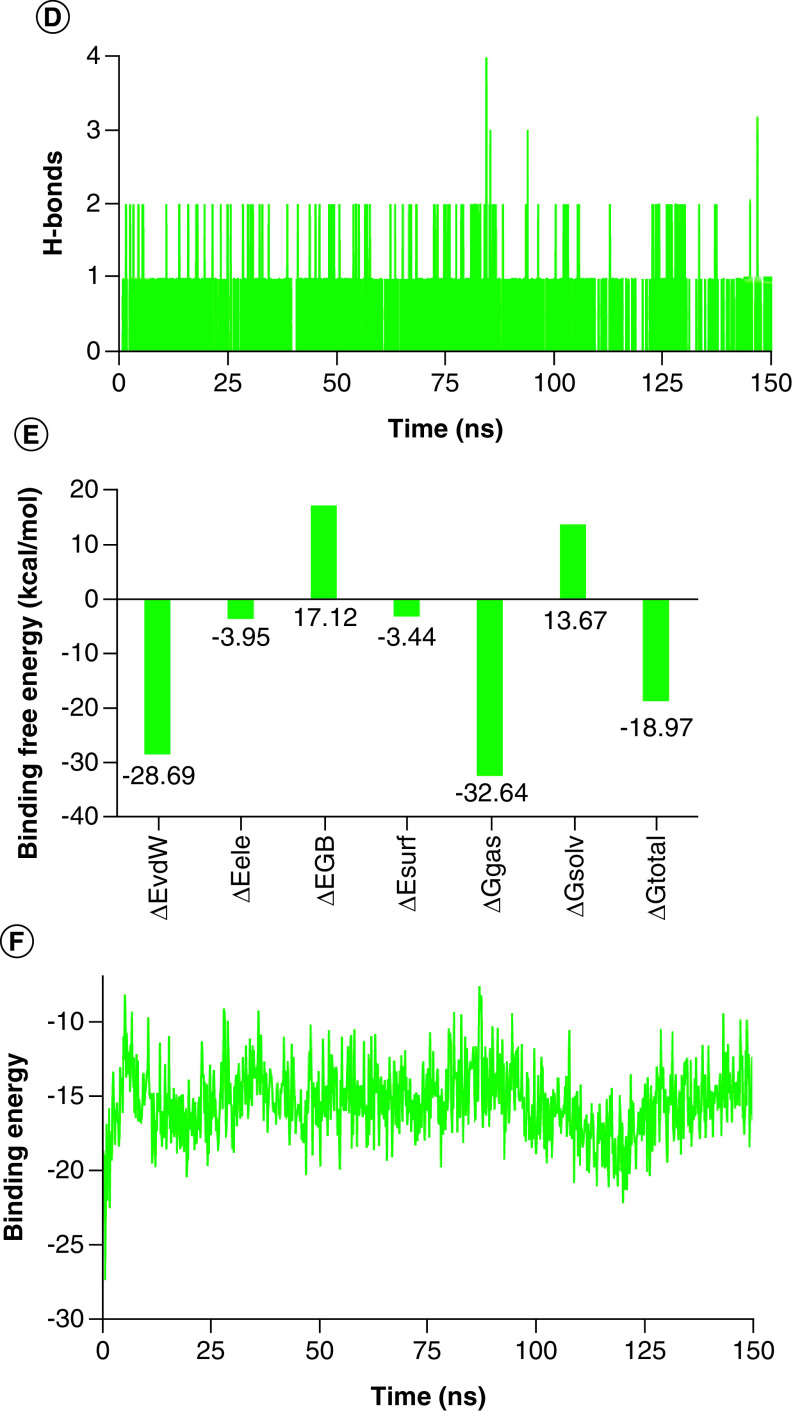
Molecular docking simulation analysis of saphenol and Omicron Spike complex. **(A)** Root mean square deviation analysis of protein backbone. **(B)** Root mean square fluctuation analysis of protein fluctuations. **(C)** Radius of gyration calculation. **(D)** Hydrogen bonding analysis. **(E)** Molecular mechanics with generalized Born and surface area solvation to calculate binding free energy. **(F)** Binding energy per frame during simulation. RMSD: Root mean square deviation; RMSF: Root mean square fluctuation.

In the saphenol and Omicron Spike complex, the RMSD of the protein backbone was equilibrated at 5 ns and then remained in the range of ∼2–3 Å till 60 ns, whereas the RMSD of apo protein showed less deviations and remained in the range of ∼2–2.5 Å (Figure 5A). The RMSF analysis revealed that the residues did not show major fluctuations in either apo protein or complex, except residues 190–200 and 320–330 (Figure 5B). The Rg values of the spike and apo protein and their complexes indicated protein compactness during simulation (Figure 5C). Therein, the formed hydrogen bonds were one or two at some frames (Figure 5D). The total binding free energy of the complex was -18.97 kcal/mol (Figure 5E), while the average energy of the complex per frame was -17 kcal/mol (Figure 5F).

### Chaetoglobosin E & Delta spike complex

**Figure 6.**
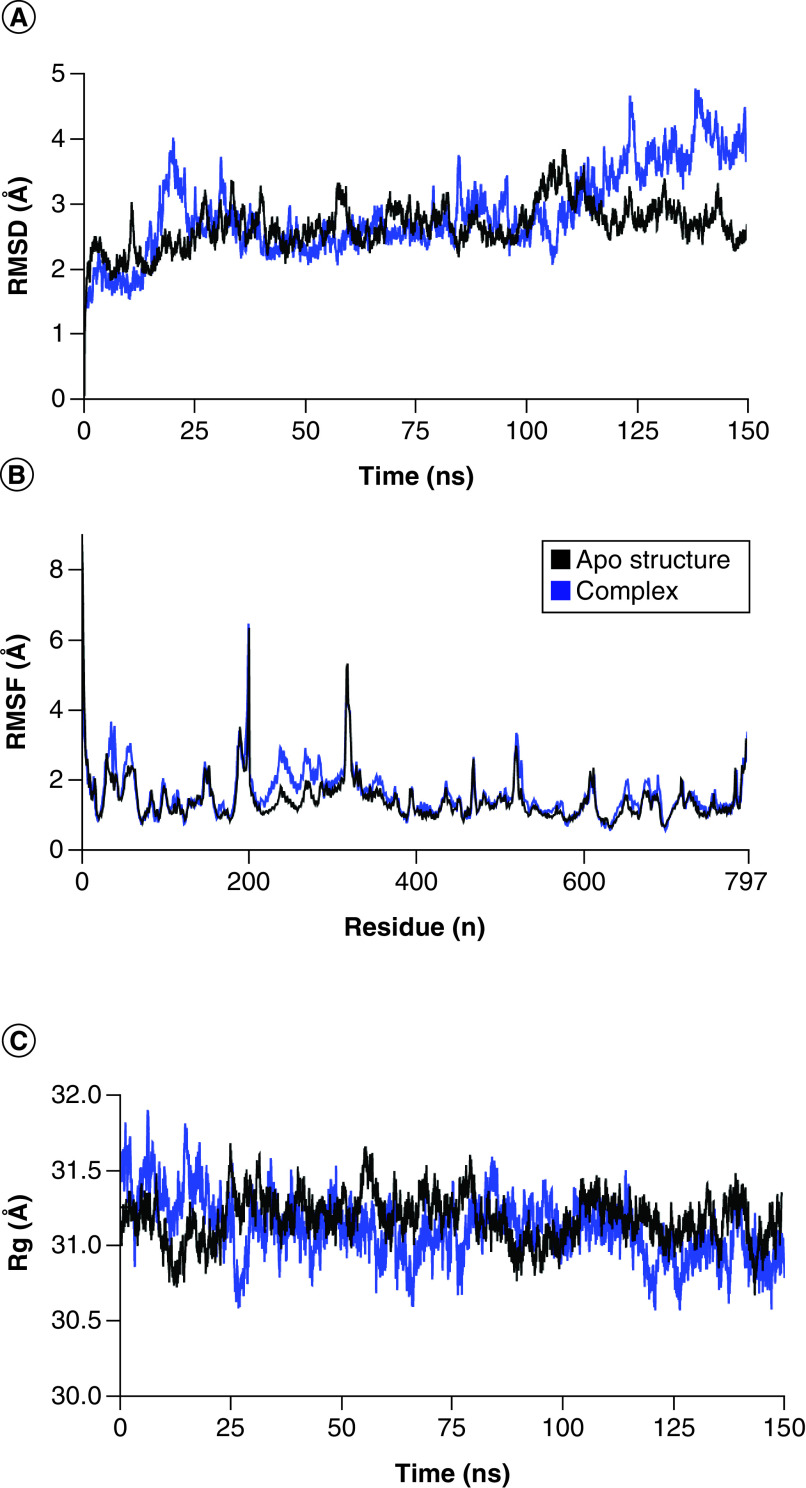

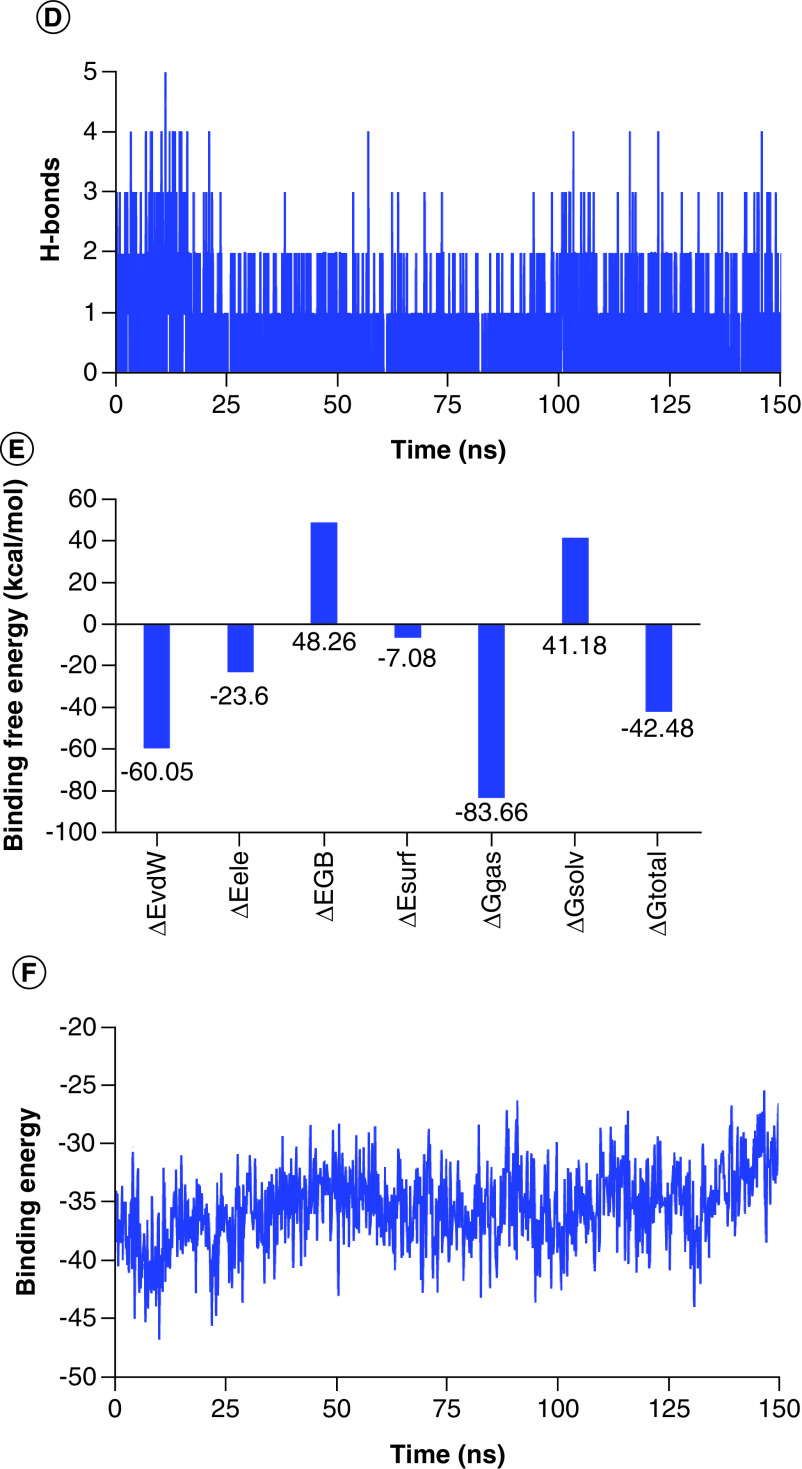
Molecular docking simulation analysis of chaetoglobosin E and Delta spike complex. **(A)** Root mean square deviation analysis of protein backbone. **(B)** Root mean square fluctuation analysis of protein fluctuations. **(C)** Radius of gyration calculation. **(D)** Hydrogen bonding analysis. **(E)** Molecular mechanics with generalized Born and surface area solvation to calculate binding free energy. **(F)** Binding energy per frame during simulation.RMSD: Root mean square deviation; RMSF: Root mean square fluctuation.

In the chaetoglobosin E and Delta Spike complex, the RMSD attained equilibrium and remained in the range of 2–4 Å between 15 and 20 ns, which however, attained stability (∼2.5 Å) till 100 ns and then gradually increased to ∼4 Å at the end of simulation (Figure 6A). Therein, RMSD of apo protein remained in the range of ∼2–3 Å. The RMSF of apo and its complex did not show major fluctuations in the residues except for residues 190–200 and 300–320 (Figure 6B). The Rg analysis of the spike and apo showed protein compactness in complex form (Figure 6C). There were 3 and 4 hydrogen bonds at some frames in the complex (Figure 6D). The total binding free energy (MMGBSA) of the complex was -42.48 kcal/mol (Figure 6E), while the average binding energy was -40 to -38 kcal/mol (Figure 6F).

## Discussion

The diminishing natural immunity and the vaccine protection challenged by the highly pathogenic SARS-CoV-2 Delta and Omicron variants due to mutations in their spike RBD and flanking region [8,34,35] have warranted the need for developing efficacious antiviral drugs. Bioinformatic tools such as molecular docking and MD simulation have been widely utilized to closely predict how a small inhibitory molecule binds with a drug target protein, establishing the ‘structure-activity relationship’. Recent *in silico* studies targeting SARS-CoV-2 spike has therefore, suggested repurposing or experimental validations of several approved drugs and bioactive phytochemicals [10–14]. In line with this, the FDA-approved anti-human immunodeficiency virus drug viracept (nelfinavir mesylate) has been recently reported to potentially inhibit SARS-CoV-2 spike mediated cell-fusion [36]. In addition, we have very recently identified natural compounds orientin, obetrioside, catechin and neridienone as strong spike-inhibitors of SARS-CoV-2 as well as its VOCs Omicron and Delta [37].

Alkaloids are a diverse class of natural organic molecules with heterocyclic tertiary nitrogen in their structure [38]. Of about 20,000 known alkaloids, most are isolated from plants and animals, including microorganisms and marine species [39]. In addition to their various pharmacological salutations, alkaloids have also been recognized for their antiviral activities against Influenza virus, herpes simplex virus, human immunodeficiency virus, murine leukemia virus, dengue virus, chikungunya virus, sindbis virus, cytomegalovirus, vesicular stomatitis virus, Ebola virus, Zika virus, hepatitis viruses, coxsackieviruses, Newcastle disease virus, enterovirus, West Nile virus, rabies virus, respiratory syncytial virus, human coronavirus, and Middle East respiratory syndrome coronavirus [40]. Notably, of the marine sources, the Caribbean deep-sea sponges, tunicates, and other species containing β-carboline alkaloids have been reported to be effective against herpes simplex virus, human immunodeficiency virus and human coronavirus [41–45]. Recently, various alkaloids and alkaloid-based drugs have been shown to have significant *in vitro* activities against SARS-CoV-2 as well as plausible effectiveness against its VOCs [46,47]. In line with this, *Camptotheca acuminata*-derived camptothecin (quinolone alkaloid) has been demonstrated to strongly inhibit SARS-CoV-2 spike binding to the ACE2 receptor [48]. In the present study, we have, for the first time, virtually screened scores of deep-sea fungal alkaloids along with viracept (ligand–control) against SARS-CoV-2, Omicron and Delta spikes, using molecular docking and MD simulation at 150 ns. Of these, *Cladosporium sphaerospermum*-derived cladosin K (tetramate alkaloid), *Cystobasidium laryngis*-derived saphenol (phenazine alkaloid) and *Chaetomium globosum*-derived chaetoglobosin E (quinoline alkaloid) were found to be the most potent inhibitors of SARS-CoV-2, Omicron and Delta spikes, respectively.

## Conclusion

Finding effective antiviral treatments has been challenging for patients with COVID-19 due to the inherent complexity of SARS-CoV-2 pathobiology, especially for its variants of concern *viz.*, Omicron and Delta. Bioinformatic-based virtual drug-discovery tools closely establish the ‘structure-activity relationship’ of a small inhibitory molecule and the target protein. Recent such studies targeting SARS-CoV-2 spike has therefore, suggested repurposing or experimental validations of several US FDA-approved drugs and bioactive natural products. Notably, the approved anti-HIV drug viracept has been recently repurposed as SARS-CoV-2 spike-inhibitor *in vitro*. In line with this, in this study, we virtually screened scores of marine fungal alkaloids, one of the most abundant bioactive natural compounds as potential source of effective and safe anti-SARS-CoV-2 therapeutics, including viracept as positive-control. We for the first time, report structure-based identification of three deep-sea fungal alkaloids cladosin K, saphenol and chaetoglobosin E as potential spike-inhibitors of SARS-CoV-2 and its Delta and Omicron variants, respectively. Because there is no evidence of natural drug-associated resistance, especially in RNA viruses, our *in silico* data warrants further experimental validations of cladosin K, saphenol and chaetoglobosin E against the Omicron and Delta spikes.

**Summary points**

- The COVID-19 pandemic caused by SARS-CoV-2 remains a global health crisis.
- Further emergences of its notable variants of concerns Delta and Omicron with increased transmissibility have also impacted vaccine efficacy.
- Deep-sea fungal alkaloids (n ≥ 150) with antiviral potential have been virtually screened for their structure-activity based activity against spike proteins of SARS-CoV-2, Delta and Omicron.
- The ligand viracept was used as positive control, while SSAYA10-001 served as a negative control.
- Following molecular docking analysis, the top compound for each target protein was chosen for the protein–ligand complex stability analysis at 150 ns molecular docking simulation.
- Their Admet scores and physiochemical properties were found to be in acceptable ranges, and they followed Lipinski's rule of five.
- Finally, three potential inhibitors *Cladosporium sphaerospermum*-derived cladosin K (tetramate alkaloid) for SARS-CoV-2, *Cystobasidium laryngis*-derived saphenol (phenazine alkaloid) for Delta, and *Chaetomium globosum*-derived chaetoglobosin E (quinoline alkaloid) for Omicron were identified.
- Our *in silico* data strongly warrants their further experimental validations against the Omicron and Delta spikes.
