# Supplementary material for: In silico identification of deep-sea fungal alkaloids as potential inhibitors of SARS-CoV-2, Delta and Omicron spikes
Source: Future Virol. 2023 Oct 26:10.2217/fvl-2023-0102. doi: 10.2217/fvl-2023-0102 (PMC10615363; doi:10.2217/fvl-2023-0102)
Supplement: Supplementary file 1 [file supplementary_data.docx]

Supplementary Table 1. Islatravir as treatment and as prevention: current and future clinical trials. When not otherwise specified, drug formulation is to be intended as "oral, tablets".

| Status | Phase; PrEP vs ART | Study title; drug | Start and end date | Study population; exclusion criteria (including, not limited to) | Sample size | location |
| --- | --- | --- | --- | --- | --- | --- |
| Active, not recruiting | Phase 3; ART | A Phase 3 Randomized, Active-Controlled, Open-Label Clinical Study to Evaluate a Switch to Doravirine/Islatravir (DOR/ISL) Once-Daily in Participants With HIV-1 Virologically Suppressed on Antiretroviral Therapy; DOR/ISL 100 mg/0.75 mg vs ART | Feb 18, 2020 – March 30, 2024 | >18-year-old, all sexes, HIV-1 RNA <50 copies/mL for ≥3 months, no history of prior virologic treatment failure; non pregnant/non breastfeeding/no hepatitis/no malignancy ≤5 years prior, not on CAB/RPV | 672 | United States, Australia, Canada, Chile, Colombia, France, Italy, Japan, New Zealand, Poland, Russian Federation, South Africa, Spain, Switzerland, United Kingdom |
| Active, not recruiting | Phase 3; ART | A Phase 3, Randomized, Active-Controlled, Double-Blind Clinical Study to Evaluate a Switch to Doravirine/Islatravir (DOR/ISL) Once-Daily in Participants With HIV-1 Virologically Suppressed on Bictegravir/Emtricitabine/Tenofovir Alafenamide (BIC/FTC/TAF); DOR/ISL 100 mg/0.75 mg and placebo to BIC/FTC/TAF vs BIC/FTC/TAF and placebo to DOR/ISL | Feb 18, 2020 – Jan 4, 2024 | >18-year-old, all sexes, HIV-1 RNA < 50 copies/mL at screening, on BIC/FTC/TAF for ≥3 months, no history of prior virologic treatment failure; non pregnant/non breastfeeding/no hepatitis/ no malignancy ≤5 years prior | 643 | United States, Australia, Austria, Canada, Finland, France, Germany, Italy, Japan, Puerto Rico, Spain |
| Recruiting | Phase 3; ART | A Phase 3 Open-label Rollover Clinical Study of Doravirine/Islatravir (DOR/ISL) Once-daily for the Treatment of HIV-1 Infection in Participants Who Previously Received DOR/ISL in a Phase 2 or Phase 3 DOR/ISL Clinical Study; DOR/ISL 100 mg/0.75 mg | Sep 15, 2021 – Oct 7, 2025 | All ages, all sexes, ≥ 35 kg, currently receiving DOR/ISL tablet in a MSD-sponsored clinical study, has completed the last treatment visit, has derived clinical benefit from DOR/ISL and further treatment with DOR/ISL is considered clinically appropriate; non pregnant/non breastfeeding | 2000 (estimated) | United States, Australia, Canada, Chile, Colombia, France, Germany, Italy, Japan, New Zealans, Poland, Russian Federation, South Africa, Spain, Switzerland, United Kingdom |
| Active, not recruiting | Phase 3; ART | A Phase 3, Randomized, Clinical Study in HIV-1-Infected Heavily Treatment-Experienced Participants Evaluating the Antiretroviral Activity of Blinded Islatravir (ISL), Doravirine (DOR), and Doravirine/Islatravir (DOR/ISL), Each Compared to Placebo, and the Antiretroviral Activity, Safety, and Tolerability of Open-Label DOR/ISL; Part 1: ISL 0.75 mg, DOR 100 mg, DOR/ISL 100 mg/0.75 mg, placebo to ISL, placebo to DOR. Part 2: DOR/ISL 100 mg/0.75 mg + optimized background therapy | Mar 18, 2020 – Oct 17 2023 | All ages, all sexes, HIV-1 positive, ≥ 35 kg, on the same ART ≥ 3 months, at least triple-class resistance, ≤2 fully active antiretroviral drugs remaining; non pregnant/non breastfeeding, no hepatitis B/other confounding conditions, not taking DOR, efavirenz, etravirine or nevirapine | 35 | United States, Australia, Canada, Chile, Colombia, France, Germany, Italy, Japan, Republic of Korea, Peru, Portugal, Puerto Rico, Russian Federation, South Africa, Spain, Ukraine, United Kingdom |
| Recruiting | Phase 3, ART | A Phase 3 Open-label Clinical Study of Doravirine/Islatravir (DOR/ISL [100 mg/0.25 mg]) Once Daily for the Treatment of HIV-1 Infection in Participants Who Previously Received DOR/ISL (100 mg/0.75 mg) QD in a Phase 3 Clinical Study; DOR/ISL 100 mg/0.25 mg | Mar 17, 2023 – Jan 14, 2026 | >18-year-old, all sexes, currently receiving DOR/ISL tablet in a MSD-sponsored clinical study; HIV-1 RNA ≥200 copies/mL in MSD DOR/ISL (100 mg/0.75 mg) MK-8591A-017 /-018 /-020, or at screening for participants entering from DOR/ISL (100 mg/0.75 mg) MK-8591A-033; CD4+ T-cell counts or lymphocyte counts in the prior DOR/ISL study that meet criteria for discontinuation; non heavily treatment-experienced | 1300 (estimated) | United States, Australia, Canada, Puerto Rico |
| Not yet recruiting | Phase 2, PrEP | A Phase 2a, Double-Blind, Placebo-Controlled Study to Evaluate the Safety, Tolerability, and Pharmacokinetics of a Radiopaque Matrix MK-8591 Implant in Participants at Low-Risk for HIV-1 Infection; ISL 47, 52 or 57 mg implantable rod (subdermal, upper arm) vs placebo | Aug 4, 2023 – Apr 2, 2025 | 18 to 55 years, all sexes, at low risk of HIV infection; non pregnant/non breastfeeding, no active hepatitis B or C/history of malignancy ≤5 years before/confounding conditions, no physical findings that interfere with implant placement | 175 (estimated) | N/A |
| Recruiting | Phase 3; ART | A Phase 3, Randomized, Active-Controlled, Open-Label Clinical Study to Evaluate a Switch to Doravirine/Islatravir (DOR/ISL 100 mg/0.25 mg) Once-Daily in Participants With HIV-1 Who Are Virologically Suppressed on Antiretroviral Therapy; DOR/ISL (100 mg/0.25 mg) vs ART | Feb 20, 2023 – Sept 5, 2025 | >18-year-old, all sexes, HIV-1 RNA < 50 copies/mL on stable ART ≥ 3 months, no history of viral failure; non pregnant/non breastfeeding, no AIDS defining infection in the last 30 days, no active hepatitis B or C/history of malignancy ≤5 years before/long-acting therapy/immunosuppressive therapy or cytochrome P450 3A inducers | 501 | United States, Australia, Canada, Japan, Switzerland, United Kingdom |
| Active, not recruiting | Phase 3, ART | A Phase 3 Randomized, Active-Controlled, Double-Blind Clinical Study to Evaluate the Antiretroviral Activity, Safety, and Tolerability of Doravirine/Islatravir Once-Daily in HIV-1 Infected Treatment-Naïve Participants; DOR/ISL (100 mg/0.75 mg) and placebo to BIC/FTC/TAF vs BIC/FTC/TAF and placebo to DOR/ISL | Feb 28, 2020 – Mar 3, 2025 | >18-year-old, HIV-1 positive, ARV naïve; non pregnant/non breastfeeding, no active hepatitis/history of malignancy ≤5 years before/confounding conditions, no virologic resistance to RTIs | 599 | United States, Argentina, Canada, Chile, Colombia, France, Germany, Israel, Italy, Japan, South Africa, Spain, Taiwan |
| Active, not recruiting | Phase 2, ART | A Phase 2b, Randomized, Active-Controlled, Double-Blind, Dose-Ranging Clinical Study to Evaluate a Switch to Islatravir (ISL) and MK-8507 Once-Weekly in Adults With HIV-1 Virologically Suppressed on Bictegravir/Emtricitabine/Tenofovir Alafenamide (BIC/FTC/TAF) Once-Daily; ISL 20 mg + Ulonivirine 100 or 200 or 400 mg and placebo to BIC/FTC/TAF vs BIC/FTC/TAF and placebo to ISL and Ulonivirine | Mar 9, 2021 – June 24, 2023 | >18-year-old, all sexes, HIV-1 RNA < 50 copies/mL, virologically suppressed on BIC/FTC/TAF ≥6 months, CD4+ T-cell count > 200/mcL; non pregnant/non breastfeeding, no active hepatitis B or C or any/history of malignancy ≤5 years prior/confounding conditions, no resistance to ulonivirine or NNRTIs | 161 | United States, France, Switzerland |
| Active, not recruiting | Phase 3, PrEP | A Phase 3, Randomized, Active-Controlled, Double-Blind Clinical Study to Evaluate the Efficacy and Safety of Oral Islatravir Once-Monthly as Preexposure Prophylaxis in Cisgender Men and Transgender Women Who Have Sex With Men, and Are at High Risk for HIV-1 Infection; monthly ISL 60 mg and daily placebo to FTC/TDF and FTC/TAF vs daily FTC/TDF or FTC/TAF and monthly placebo to ISL | Mar 15, 2021 – Sep 27, 2024 | >16, assigned male at birth, HIV-uninfected, has anal sexual intercourse with a man or transgender woman at least once in the past month, at high risk for HIV-1 infection; no active hepatitis B or liver disease/history of malignancy ≤5 years prior/confounding conditions, no prior long-acting PrEP exposure | 494 | United States, Brazil, France, Japan, Peru, South Africa, Thailand |
| Active, not recruiting | Phase 3, PrEP | A Phase 3, Randomized, Active-Controlled, Double-blind Clinical Study to Evaluate the Efficacy and Safety of Oral Islatravir Once-Monthly as Preexposure Prophylaxis in Cisgender Women at High Risk for HIV-1 Infection; monthly ISL 60 mg and daily placebo to FTC/TDF vs daily FTC/TDF and monthly placebo to ISL | Feb 24, 2021 – July 5, 2024 | 16 to 45, assigned female at birth, HIV uninfected, sexually active at high risk for HIV-1 infection; non pregnant/non breastfeeding, no hepatitis B/liver disease history of malignancy ≤5 years prior, no prior long-acting PrEP exposure | 730 | United States, South Africa, Uganda |
| Recruiting | Phase 2, ART | A Phase 2 Randomized, Open-Label, Active-Controlled Study Evaluating the Safety and Efficacy of an Oral Weekly Regimen of Islatravir in Combination With Lenacapavir in Virologically Suppressed People With HIV; ISL 2 mg + LEN 600 mg on day 1, LEN 600 mg on day 2, then weekly ISL 2 mg and LEN 300 mg vs BIC/FTC/TAF | Oct 5, 2021 – Dec 2027 | >18-year-old, all sexes, HIV-1 RNA 50 copies/mL at screening, virologically suppressed on BIC/FTC/TAF for ≥ 24 weeks at screening; non pregnant/non breastfeeding, no history of virologic failure, no prior exposure to ISL or LEN, no serious infections in last 30 days/hepatitis B or C/creatinine clearance < 30 mL/min | 136 (estimated) | United States |
| Recruiting | Phase 3, ART | A Phase 3, Randomized, Active-Controlled, Double-Blind Clinical Study to Evaluate a Switch to Doravirine/Islatravir (DOR/ISL 100 mg/0.25 mg) Once-Daily in Participants With HIV-1 Who Are Virologically Suppressed on Bictegravir/Emtricitabine/Tenofovir Alafenamide (BIC/FTC/TAF); DOR/ISL (100 mg/0.25 mg) and placebo to BIC/FTC/TAF vs BIC/FTC/TAF and placebo to DOR/ISL | Fen 17, 2023 – Aug 15, 2025 | >18-year-old, all sexes, HIV-1 RNA < 50 copies/mL, virologically suppressed on BIC/FTC/TAF ≥ 3months, no history of prior virologic failure; non pregnant/non breastfeeding, no AIDS-defining opportunistic infection in the past 30 days, no active hepatitis B or C/history of malignancy ≤5 years prior, no immunosuppressive therapy or cytochrome P450 3A inducers, no resistance to DOR, no long-acting exposure | 501 (estimated) | United States, Australia, Israel, Japan, United Kingdom |
| Recruiting | Phase 3, ART | A Phase 3, Randomized, Active-Controlled, Double-Blind Clinical Study to Evaluate the Antiretroviral Activity, Safety, and Tolerability of Doravirine/Islatravir (DOR/ISL 100 mg/0.25 mg) Once-Daily in HIV-1 Infected Treatment-Naïve Participants; DOR/ISL (100 mg/0.25 mg) and placebo to BIC/FTC/TAF vs BIC/FTC/TAF and placebo to DOR/ISL | March 8, 2023 – July 31, 2026 | >18-year-old, all sexes, HIV-1 RNA > 500 copies/mL, ART naïve; non pregnant/non breastfeeding, no AIDS defining opportunistic infections in the last 30 days/no hepatitis B or C/no history of malignancy ≤5 years prior/no confounding conditions | 500 (estimate) | United States, Israel, Japan, Puerto Rico |
